# 

**Published:** 1918-01-05

**Authors:** 


RATES OF SUBSCRIPTION: Twelve months, 6/6; Six months, 4/-; three months, 2/-, post free to any address in the United Kingdom. Abroad. Twelve months, 8/8; Six months, 5/-; Three months, 2/6, post free. THE HOSPITAL "Index" to Volume LXII., April 1917 to September 1917, is now ready, price 2½d. per copy, post free. Address The Manager, THE HOSPITAL, "The Hospital" Building, 28/29 Southampton Street, Strand, London, W.C. 2.

### Recently Published Parliamentary Returns, etc.

Ministry of Pensions. Memorandum on the Functions and Powers of the Pensions Appeal Tribunal. Post free 1½d.

National Health Insurance. Medical Benefit Regulations (Wales). Provisional Regulations, November 23, 1917, made by the Joint Committee acting jointly with the Welsh Insurance Commission under Section 15 of the National Insurance Act, 1911. Post free 1½d.

National Health Insurance. Medical Benefit Regulations, 1917. Provisional Regulations, November 21, 1917. Post free 1½d.

National Health Insurance. Medical Benefit Regulations, 1917. Draft Regulations, November 21, 1917. Post free 1½d.

Pauperism (England and Wales). Quarterly Statements to September 1917. Post free 4d.

Report from Standing Committee "A" on the National Health Insurance Bill, with the Proceedings of the Committee. Post free 3d.

National Insurance Acts, 1911-1917. Return as to the administration of Sanatorium Benefit from January 12, 1914, to December 31, 1914, and January 1, 1915, to December 31, 1915. Post free 1½d.

National Health Insurance. Medical Benefit Regulations (Scotland), 1917, November 21, 1917. Post free 1½d.

National Health Insurance Bill. Post free 9d.

The above may be obtained from H.M. Stationery Office, Imperial House, Kingsway, W.C. 2.

### Forthcoming Meetings.

A Trip to America and American Hospitals.—An illustrated lecture on this subject will be given, under the auspices of the Royal British Nurses' Association, by Mr. Herbert Paterson, M.C., F.R.C.S., on Thursday, January 10, at 2.45 p.m., at the Robert Barnes Hall, 1 Wimpole Street, Cavendish Square, W., H.E. the American Ambassador in the chair.

